# Angiotensin II‐induced natriuresis is attenuated in knockout mice lacking the receptors for tumor necrosis factor‐α

**DOI:** 10.14814/phy2.14942

**Published:** 2021-08-02

**Authors:** Dewan S. A. Majid, Alexander Castillo

**Affiliations:** ^1^ Department of Physiology Tulane Hypertension & Renal Center of Excellence Tulane University Health Sciences Center New Orleans LA USA

**Keywords:** angiotensin II, natriuresis, renal function, TNF‐α, TNF‐α receptors

## Abstract

Intravenous infusion of relatively higher doses of angiotensin II (AngII) elicits natriuresis as opposed to its usual anti‐natruretic response. As AngII can induce tumor necrosis factor‐α (TNFα) production which elicits natriuresis via its action on TNFα receptor type 1 (TNFR1), we hypothesize that the concomitant release of TNFα contributes to the natriuretic response to AngII. Responses to AngII infusion (1 ng min^−1^ g^−1^ for 75 min, iv) were evaluated in anesthetized knockout (KO) mice lacking TNFR1 (*n* = 6) and TNFR2 (TNFα receptor type 2; *n* = 6) and compared these responses with those in wild type (WT; *n* = 6) mice. Arterial pressure (AP) was recorded from a cannula placed in the carotid artery. Renal blood flow (RBF) and glomerular filtration rate (GFR) were measured by PAH and inulin clearances, respectively. Urine was collected from a catheter placed in the bladder. AngII caused similar increases (*p* < 0.05 vs basal values) in AP (WT, 37 ± 5%; TNFR1KO, 35 ± 4%; TNFR2KO, 30 ± 4%) and decreases (*p* < 0.05) in RBF (WT, −39 ± 5%; TNFR1KO, −28 ± 6%; TNFR2KO, −31 ± 4%) without significant changes in GFR (WT, −17 ± 7%; TNFR1KO, −18 ± 7%; TNFR2KO, −12 ± 7%). However, despite similar changes in AP and renal hemodynamics, AngII induced increases (*p* < 0.05) in urinary sodium excretion in WT (3916 ± 942%) were less in the KO strains, more or less in TNFR1KO (473 ± 170%) than in TNFR2KO (1176 ± 168%). These data indicate that TNF‐α receptors, particularly TNFR1 are involved in the natriuretic response that occur during acute infusion of AngII and thus, plays a protective role in preventing excessive salt retention at clinical conditions associated with elevated AngII level.

## Graphical abstract

Short summary of the study: The present study examined the role of concomitant TNF‐α formation in regulating renal responses to angiotensin II (AngII) by evaluating its effects in mice lacking TNF receptor type 1 (TNFR1KO) and type 2 (TNFR2KO) and compared to that in WT mice. The results showed that TNFR1 activation is involved in the natriuresis induced by AngII and suggest a protective role of TNFR1 in preventing excessive salt retention during elevated AngII level.

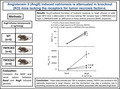

## 1 INTRODUCTION

Different studies have implicated an involvement of tumor necrosis factor‐alpha (TNF‐α), a pro‐inflammatory cytokine, in the pathophysiology of angiotensin II (AngII)‐dependent hypertension and the development of renal injury (Elmarakby et al., 2006; Guzik et al., 2007; Mehaffey & Majid, 2017; Nguyen et al., 2013; Satou et al., 2018; Zhang et al., 2014). Since AngII treatment induces the production of TNF‐α in the kidney (Rodriguez‐Iturbe et al., 2014; Ruiz‐Ortega et al., 2002; Satou et al., 2018), a functional cross‐talk between AngII and TNF has been suggested in many renal pathophysiological processes (Majid et al., 2015; Mehaffey & Majid, 2017). Involvement of TNF‐α mediated inflammatory process in AngII‐induced renal pathophysiology is usually a chronic event, as this cytokine also regulates production of other cytokines, chemokines, and adhesion molecules that are involved in prolonged cellular responses, including cell proliferation and differentiation of inflammatory cells leading to renal injury and fibrosis resulting progression of chronic kidney disease (Mehaffey & Majid, 2017; Satou et al., 2018; Zhang et al., 2014). However, the functional role of TNF‐α in AngII‐induced acute responses in the kidney is not yet clearly defined.

It is well recognized that an enhanced renin–angiotensin system (RAS) stimulates the production of many pro‐inflammatory cytokines including TNF‐α in the kidney (Guzik et al., 2007; Ruiz‐Ortega et al., 2002; Zhang et al., 2014). Thus, it is important to know the contribution of this concomitant TNF‐α formation in the renal vascular and tubular functional responses to elevated RAS. It is well known that AngII activates the immune systems, leading to infiltration of T lymphocytes into the kidney and vasculature that produces TNF‐α (Guzik et al., 2007; Zhang et al., 2014), though the minimum time required to produce TNF by AngII treatment is not clearly demonstrated in those studies. However, previous studies have reported a significant increase in TNF‐α mRNA and protein biosynthesis in a time period as short as 30–60 min in response to AngII treatment in isolated buffer‐perfused Langendorff feline hearts as well as in cultured adult cardiac myocytes and these responses were mediated through the angiotensin type 1 receptor (Kalra et al., 2002). We have also reported that the TNF‐α production and its plasma level increased within 60 min of systemic infusion of nitric oxide (NO) synthase inhibitor in mice (Shahid et al., 2010). Our earlier studies have demonstrated that the infusion of TNF‐α in mice induces a marked natriuretic response within 60 min of its infusion though it causes renal vasoconstrictor and glomerular hypofiltration effects (Shahid et al., 2008). However, the contribution of TNF‐α and its receptors subtypes, TNF‐α receptors type 1 (TNFR1) and type 2 (TNFR2) in the renal hemodynamic and excretory functions, particularly in this natriuretic response to acute AngII infusion is not yet clearly determined.

In the present study, we have examined the hypothesis that a concomitant generation of TNF‐α during AngII administration contributes to the renal vascular as well as tubular actions of AngII by a differential activation of TNFR1 and TNFR2 in the kidney (Battula et al., 2011; Mehaffey & Majid, 2017; Singh et al., 2013). To examine this hypothesis, the responses to intravenous infusion of AngII were evaluated in knockout mice lacking TNFR1 (TNFR1KO) and TNFR2 (TNFR2KO) to assess the contribution of TNF‐α in the renal actions of AngII. Acute infusion of a relatively higher dose of AngII (1 ng min^−1^ g^−1^ for 30 min, iv) was given in anesthetized TNFR1KO and TNFR2KO mice and the responses were compared with those in wild‐type (WT) mice.

## 2 METHODS

All the experimental procedures were approved by and performed in accordance with the guidelines and practices established by the Tulane University Animal Care and Use Committee.

Male mice in which the TNF‐α receptors TNFR1 (B6.129‐Tnfrsf1atm1Mak/J) and TNFR2 (B6.129S2‐Tnfrsf1btm1Mwm/J) had been genetically knocked out and their background WT (WT; C57BL/6) mice were used in these experiments (Jackson Laboratories). All these mice (*n* = 6 in each group; 9–10 week old with average body weights, WT, 25.7 ± 0.7 g; TNFR1KO, 24.2 ± 0.8 g; TNFR2KO, 24.9 ± 0.6 g) were purchased from Jackson Laboratories. Male mice were only used in this study as we have conducted similar acute experiments in our earlier studies which give us the advantage to compare these results with findings from other similar studies. (Castillo et al., 2012; Shahid et al., 2008). These mice were housed in a temperature‐ and light‐controlled room in the Tulane Vivarium and allowed free access to standard diet (Ralston‐Purina) and tap water for ≥3 days before acute renal clearance studies were performed under anesthesia.

### 2.1 Surgical preparation

On the day of experiments, the mice were anesthetized with inactin (150 mg/kg, i.p.), and the renal clearance studies were performed as described previously (Castillo et al., 2012; Shahid et al., 2008). The mice were placed on a servo‐controlled surgical table that maintained body temperature at 37℃, and a tracheostomy was performed. The mice were given breathing air enriched with O2: the exterior end of the tracheal cannula was placed inside a small plastic chamber into which humidified 95% O_2_‐5% CO_2_ was continuously passed. The right carotid artery was cannulated with polyethylene (PE‐10) tubing connected to a pressure transducer (AcqKnowledge data acquisition system, Biopac) for continuous recording of mean arterial pressure (MAP) and heart rate. The carotid artery cannula tip was advanced up to the arch of aorta and thus, MAP was equal to aortic pressure in these preparations. The right jugular vein was catheterized with PE‐10 tubing for isotonic saline (0.9% NaCl) infusion at a rate of 3 μl/min with the help of an infusion pump (CMA). During surgery, an isotonic saline solution containing 6% albumin (bovine serum; Calbiochem) was infused. The bladder was catheterized with PE‐90 tubing via a suprapubic incision for urine collection. After surgery, the infusion fluid was replaced with isotonic saline solution containing 1% albumin, 7.5% inulin (Inutest, Laevosan), and 1.5% PAH (Merck Sharpe & Dohme), which was also used as a vehicle for AngII solution. Inulin and PAH solutions are used to measure their clearances to determine glomerular filtration rate (GFR) and renal blood flow (RBF), respectively.

### 2.2 Experimental protocol

The basic protocol with infusion of AngII in all the experiments in mice was as follows. After a 60‐min equilibration period following completion of surgical procedures, two consecutive 30‐min control urine collections (basal period) were performed. An arterial blood sample (100 μl) was taken for measurements of basal hematocrit and plasma PAH, inulin, and sodium/potassium concentrations. Then an infusion of AngII was started at a rate of 1.0 ng g^−1^ min^−1^ and continued until the end of the experiment. After a stabilization period of 15 min after the initiation of AngII infusion, another two 30‐min urine clearance collections were performed (treatment period). After the final collection period, another arterial blood sample (100 μl) was taken for measurements of hematocrit, plasma PAH, inulin, and sodium/potassium concentrations. At the end of the experiment, the animals were euthanized with a high dose of anesthetic, and the kidneys were removed and weighed.

### 2.3 Calculation and statistical analysis

Urine flow (V) was measured gravimetrically. Blood and urine samples collected during experimental periods were analyzed for inulin, PAH, and sodium/potassium concentrations, as reported earlier (Castillo et al., 2012; Singh et al., 2014). The collected blood samples were centrifuged to obtain plasma samples, which were used for these analyses. Inulin and PAH concentrations were determined by spectrophotometry, and sodium/potassium concentrations were determined by flame photometry. Inulin clearance is considered for glomerular filtration rate (GFR), and PAH clearance is considered for renal plasma flow (RPF). RBF is calculated from RPF and hematocrit. The concentration of sodium in urine and blood is used to calculate urinary sodium excretion rate (U_Na_V) and fractional excretion of sodium (FE_Na_), respectively. Renal vascular resistance (RVR) is calculated by dividing mean arterial pressure (MAP) by RBF. The mean of the values obtained during the first two control collection periods was considered the “basal value,” while the mean of the values collected during the two collection periods during AngII infusion was taken as the “treatment value.” The differences in the values between the basal and treatment periods are considered as the responses to AngII treatment. All values were normalized per gram (g) of kidney weight (average kidney weights as follows: WT, 0.31 ± 0.08 g; TNFR1KO, 0.34 ± 0.11 g; TNFR2KO, 0.33 ± 0.10 g). Results are expressed as means ± *SE*. Statistical differences between the basal and AngII treatment period values in the same group of mice (*n* = 6 in each group) were analyzed using paired Student's *t*‐test. However, the responses (differences in the basal and treatment period values) in the TNFR1KO or TNFR2KO groups were compared with those in the WT group (*n* = 6 in each group) using one‐way ANOVA analysis. The same statistical method was also used to compare the basal values at different parameters obtained from different groups. Differences are considered significant at *p* < 0.05.

## 3 RESULTS

**FIGURE 1:**
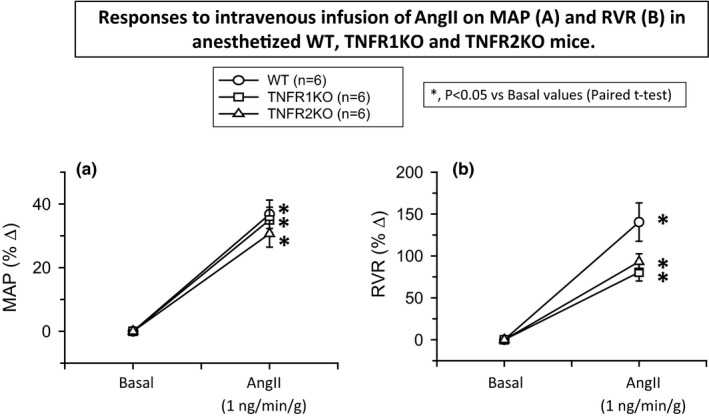
Responses to intravenous infusion of angiotensin II (AngII; 1 ng min^−1^ g^−1^ bw) on mean arterial pressure (MAP; a) and renal vascular resistance (RVR; b) in wild‐type mice (WT, *n* = 6), TNF receptor type 1 knockout mice (TNFR1KO, *n* = 6) and TNF receptor type 2 knockout mice (TNFR2KO, *n* = 6). Statistical differences between the basal and AngII treatment period values are analyzed by paired Student's *t*‐test (Significant difference is denoted by *) and the comparison of the responses in WT with TNFR1KO and TNFR2KO mice are compared by one‐way ANOVA (significant difference is denoted by #)

**FIGURE 2:**
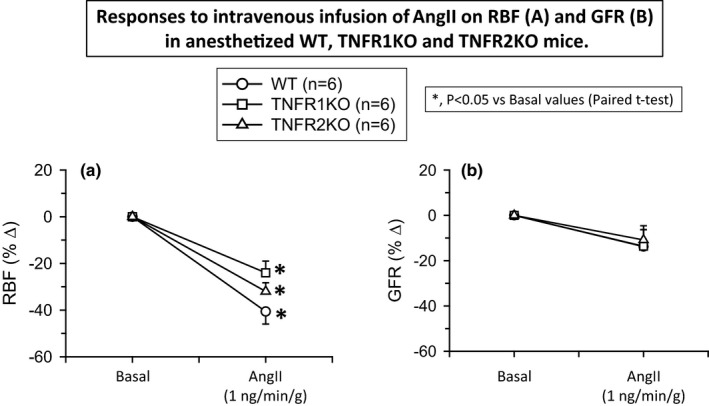
Responses to intravenous infusion of angiotensin II (AngII; 1 ng min^−1^ g^−1^ bw) on renal blood flow (RBF; a) and glomerular filtration rate (GFR; b) in wild‐type mice (WT, *n* = 6), TNF receptor type 1 knockout mice (TNFR1KO, *n* = 6) and TNF receptor type 2 knockout mice (TNFR2KO, *n* = 6). Statistical differences between the basal and AngII treatment period values are analyzed by paired Student's *t*‐test (significant difference is denoted by *) and the comparison of the responses in WT with TNFR1KO and TNFR2KO mice are compared by one‐way ANOVA (significant difference is denoted by #)

**FIGURE 3:**
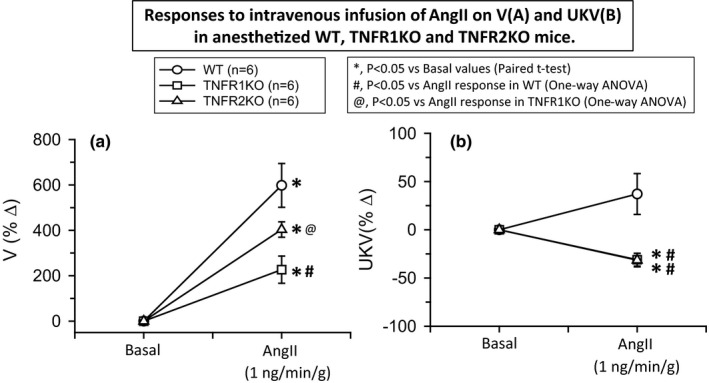
Responses to intravenous infusion of angiotensin II (AngII; 1 ng min^−1^ g^−1^ bw) on urine flow (V; a) and urinary potassium excretion (UKV; b) in wild‐type mice (WT, *n* = 6), TNF receptor type 1 knockout mice (TNFR1KO, *n* = 6) and TNF receptor type 2 knockout mice (TNFR2KO, *n* = 6). Statistical differences between the basal and AngII treatment period values are analyzed by paired Student's *t*‐test (significant difference is denoted by *) and the comparison of the responses in WT with TNFR1KO and TNFR2KO mice are compared by one‐way ANOVA (Significant difference is denoted by # and @)

**FIGURE 4:**
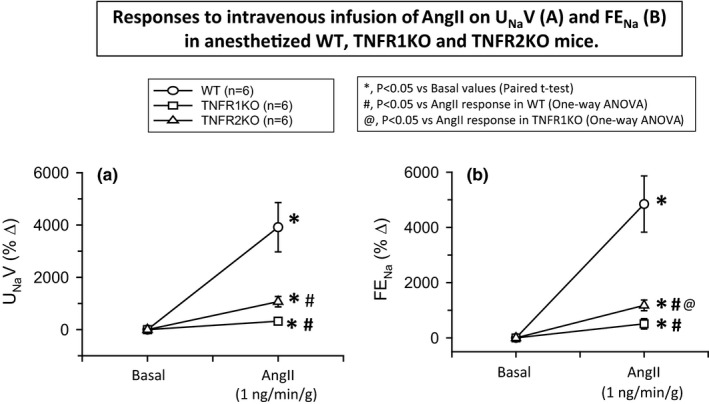
Responses to intravenous infusion of angiotensin II (AngII; 1 ng min^−1^ g^−1^ bw) on urinary sodium excretion (U_Na_V; a) and fractional excretion of sodium (FE_Na_; b) in wild‐type mice (WT, *n* = 6), TNF receptor type 1 knockout mice (TNFR1KO, *n* = 6) and TNF receptor type 2 knockout mice (TNFR2KO, *n* = 6). Statistical differences between the basal and AngII treatment period values are analyzed by paired Student's *t*‐test (significant difference is denoted by *) and the comparison of the responses in WT with TNFR1KO and TNFR2KO mice are compared by one‐way ANOVA (significant difference is denoted by # and @)

The mean values in each group (WT, TNFR1KO, and TNFR2KO) of mice obtained during the basal and treatment periods with AngII infusion are given in Table 1. The differences in the basal and treatment period values were considered as the response to AngII infusion. The basal values of all parameters in these three groups of mice were not statistically different except in sodium excretion values which showed a significantly higher level in TNFR1KO compared to that in WT mice (Table 1). As the basal values varies in these mice, the responses in all these three groups of animals were normalized by calculating the percentage changes in each animal. These normalized responses are illustrated in Figures 1, 2, 3, 4.

**TABLE 1:** Renal responses to intravenous infusion of angiotensin II (AngII; 1 ng min^−1^ g^−1^ bw)

Parameters	WT (*n* = 6) Basal period—treatment period	TNFR1KO (*n* = 6) Basal period—treatment period	TNFR2KO (*n* = 6) Basal period—treatment period
Mean arterial pressure (mmHg)	92 ± 4–125 ± 6*	82 ± 2–110 ± 5*	90 ± 4–116 ± 3*
Renal blood flow ( ml min^−1^ g^−1^ kw)	7.3 ± 0.7–4.2 ± 0.1*	6.8 ± 0.7–4.9 ± 0.6*	6.0 ± 0.2–4.0 ± 0.2*
Renal vascular resistance (mmHg ml^−1^ min^−1^ g^−1^ kw)	12.9 ± 0.8–30.2 ± 1.4*	11.7 ± 1.8–24.7 ± 3.0*	15.4 ± 0.8–29.6 ± 1.9*
Glomerular filtration rate ( ml^−1^ min^−1^ g^−1^ kw)	1.2 ± 0.1–1.0 ± 0.02	1.6 ± 0.1–1.2 ± 0.1	1.5 ± 0.1–1.3 ± 0.1
Urine flow ( ml^−1^ min^−1^ g^−1^ kw)	5.02 ± 0.4–34.3 ± 4.4*	9.5 ± 1.5–22.5 ± 4.8*	6.0 ± 0.6–26.7 ± 1.4*
Sodium excretion ( ml^−1^ min^−1^ g^−1^ kw)	0.18 ± 0.2–6.3 ± 0.8*	1.4 ± 0.2^#^–3.8 ± 0.6*	0.4 ± 0.1–4.5 ± 0.4*
Fractional excretion of sodium (%)	0.11 ± 0.02–4.7 ± 0.6*	0.6 ± 0.3–2.1 ± 0.4*	0.22 ± 0.04–2.4 ± 0.3*
Potassium excretion ( ml^−1^ min^−1^ g^−1^ kw)	1.0 ± 0.1–1.3 ± 0.1	1.1 ± 0.1–0.7 ± 0.1*	1.2 ± 0.1–0.8 ± 0.1*

Note

Statistical analysis by paired *T*‐test.

Abbreviations: Kw, kidney weight; TNFR1KO, TNF receptor type 1 Knockout mice; TNFR2KO, TNF receptor type 2 Knockout mice; WT, wild type.

* *p* < 0.05 vs basal values.

# *p* < 0.05 vs basal values in WT.

### 3.1 Effect of AngII infusion on MAP in TNFR1KO and TNFR2KO mice

The mean MAP values during the basal and treatment periods recorded in these three groups of mice (*n* = 6 in each group) are given in Table 1. AngII infusion in these groups of mice induces similar increases in MAP in TNFR1KO (38 ± 4%) and in TNFR2KO (31 ± 4%). These responses were not significantly different from that observed in WT mice (37 ± 5%) when compared using one‐way ANOVA analysis. Figure 1A illustrates these normalized values (percent changes) of the responses to AngII on MAP in these three groups of mice.

### 3.2 Effect of acute AngII infusion on renal hemodynamics in TNFR1KO and TNFR2KO mice

The mean values of RBF, RVR, and GFR during the basal and treatment periods in these three groups of mice are given in Table 1. The mean basal values in these groups of mice were not statistically different. AngII infusion in WT mice decreased RBF (−41 ± 5%) and increased RVR (140 ± 23%). A similar qualitative response in RBF and RVR was observed in TNFR1KO (RBF, −27 ± 6% and RVR, 99.5 ± 22%) and in TNFR2KO mice (RBF, −31 ± 4% and RVR, 93 ± 9%). Although the AngII‐induced responses seem less in both KO strains compared to those in WT, these differences are not statistically significant when compared using one‐way ANOVA analysis (Tukey's test). The p‐value of the RBF responses between WT and TNFR1KO is 0.334 and between WT and TNFR2KO is 0.283. Similarly, the *p*‐value sof the RVR responses between WT and TNFR1KO is 0.218 and between WT and TNFR2KO is 0.113. However, AngII administration did not cause any significant change in GFR in WT or in TNFR1KO and TNFR1KO mice. The normalized responses (percent changes) to AngII in RVR, RBF, and GFR are illustrated in Figures 1B and 2A,B respectively.

### 3.3 Effect of acute AngII infusion on renal excretory function in TNFR1KO and TNFR2KO mice

The mean values of V, U_Na_V, FE_Na_, and UKV during the basal and treatment periods in these three groups of mice are given in Table 1. The basal level of these excretory parameters in these three groups of mice were not statistically different except in U_Na_V values which showed a significantly higher level in TNFR1KO compared to that in WT mice. In WT mice, this AngII dose caused massive increases in V (598 ± 97%; *p* < 0.001), U_Na_V (3916 ± 942%; *p* < 0.001), and FE_Na_ (4886 ± 1018%; *p* < 0.001), but no statistical changes in UKV (43 ± 19%). However, these AngII infusion responses in V, U_Na_V, and FE_Na_ were much reduced in magnitude in TNFR1KO mice than that in TNFR2KO mice. The percent changes in the renal excretory responses (V, U_Na_V, FE_Na_, and UKV) to AngII infusion in TNFR1KO and TNFR2KO as well as in WT mice are shown in Figures 3A,B and 4A,B. In TNFR1KO mice, AngII infusion caused an increase in V (227 ± 60%; *p* < 0.05; Figure 3A), U_Na_V (473 ± 170%; *p* < 0.05; Figure 4A), and FE_Na_ (581 ± 184%; *p* < 0.05; Figure 4B) but slightly decreased in UKV (−31 ± 7%; *p* < 0.05; Figure 3B). In TNFR2KO mice, AngII infusion caused an increase in V (404 ± 34%; *p* < 0.01; Figure 3A), in U_Na_V (1176 ± 168%; *p* < 0.01; Figure 4A) and FE_Na_ (1313 ± 172%; *p* < 0.01; Figure 4B), but slightly decreased in UKV (−32 ± 7%; *p* < 0.01). However, when the differences in the V, U_Na_V, and FE_Na_ responses in the three groups were analyzed using one‐way ANOVA (Tukey's test), it was noted that only U_Na_V and FE_Na_ responses in TNFR1KO and TNFR2KO were reduced significantly (*p* < 0.05) when compared with that in WT whereas the V response in TNFR2KO was not that significant (*p* = 0.096) when compared with that in WT. Moreover, the differences in the V and FE_Na_ responses between the TNFR1KO and TNFR2KO groups were significantly (*p* < 0.05) different but the U_Na_V response while approaching the significance level (*p* = 0.065) was not significant. Anyway, the differences in UKV responses both in the TNFR1KO and TNFR2KO groups were significantly different (*p* < 0.05) than that in WT mice.

### 3.4 Results in time‐control experiments

In the time–control experiments (*n* = 4), no significant difference was observed between the values obtained from the basal and the treatment collection periods, which were as follows: RBF, 5.7 ± 0.6 to 5.9 ± 0.7 ml min^−1^ g^−1^; GFR, 0.65 ± 0.07 to 0.68 ± 0.09 ml min^−1^ g^−1^; V, 6.6 ± 0.8 to 9.6 ± 1.8 μl min^−1^ g^−1^; and U_Na_V, 0.42 ± 0.02 to 0.72 ± 0.21 μmol min^−1^ g^−1^; FE_Na_, 0.43 ± 0.04 to 0.77 ± 0.25%, and UKV, 1.2 ± 0.3 to 1.4 ± 0.1 μmol min^−1^ g^−1^. These findings indicate that the possibility of time‐dependent changes in urine flow or renal excretory parameters in these protocol periods are minimal in the present study as shown also in our earlier studies (Castillo et al., 2012; Shahid et al., 2008).

## 4 DISCUSSION

In this investigation, it has been demonstrated that the diuretic and natriuretic responses that usually occur during acute administration of a pressor dose of AngII in WT mice are attenuated in knockout stains of mice lacking both types TNF‐α receptors‐ more markedly attenuated in TNFR1KO than in TNFR2KO. Such attenuation of sodium excretory function occurs despite no comparable changes in arterial pressure and in renal hemodynamic parameters in response to AngII infusion in these three strains (WT, TNFR1KO, and TNFR2KO) of mice. In the kidney, AngII usually stimulates renal tubular water and salt reabsorption causing anti‐natriuresis and anti‐diuresis (Majid et al., 2015; Satou et al., 2018) via enhancing multiple sodium transport activities, particularly enhancing the activity of the epithelial Na^+^ channel (ENaC) in the aldosterone‐sensitive distal nephron (Mamenko et al., 2012). However, while it is clearly recognized that the direct effect of AngII on renal tubule is that of an anti‐natriuretic effect (Mamenko et al., 2012; Zaika et al., 2013), a paradoxical natriuretic response to systemic administration of a pressor dose of AngII was not yet clearly understood. AngII infusions like these in the present study are mostly used to mimic the responses that would occur when there is inappropriate stimulation of the RAS as in hypertension or with renal arterial stenosis. The estimated renal plasma concentration of AngII achieved during the acute infusion period of AngII dose in the present study was ∼1500 fmol/ml, which was somewhat higher than that observed in chronic AngII‐induced hypertensive mice (Gonzalez‐Villalobos et al., 2008) and also approximately 10 times higher than the baseline values reported in C57BL6 mouse (Wysocki et al., 2015). So, this AngII infusion would achieve a pathophysiological level and the findings in response to this dose are relevant to the hypertensive conditions with enhanced RAS stimulation.

It was suggested earlier that such natriuretic responses could be related to increases in arterial pressure in response to pressor doses of AngII administration (Nguyen et al., 2013, 2015; Zhao & Navar, 2008) but the mechanism was not clearly explained as some studies suggested that it is caused due to changes in sodium reabsorption in proximal tubule (Nguyen et al., 2013, 2015) while the other suggested that such changes occur in distal tubule (Zhao & Navar, 2008). In the present investigation, it has been shown that AngII administration at the pressor dose caused similar increases in MAP in these three strains of mice but the natriuretic response is different between these strains with marked attenuation observed in TNFR1KO and less insignificant change in TNFR2KO, compared to that in WT mice. Renal vasoconstrictor response to AngII dose is also similar in these knockout strains which are not statistically different to that in WT mice. Thus, these findings suggest that AngII‐induced natriuretic response is not simply related to the increases in arterial pressure or changes in the filtered tubular load of sodium, but to the other factor(s) associated with such infusion of AngII. As the natriuretic response is attenuated in mice lacking TNF‐α receptors, these data strongly suggest that TNF‐α is mechanistically involved, at least partially, in inducing such enhanced sodium excretory function of AngII.

It may be argued that these differences in the renal responses to AngII in these three groups of animals may be related to the changes in the renal arterial pressure (RAP) responses as reported in an earlier study using dog preparation (Olsen et al., 1985) which had employed a servo‐control device to mechanically reduce the RAP. While such mechanical reduction in RAP obviously affected the tubular excretory function to AngII in that preparation (Olsen et al., 1985), such findings could not be correlated with our present findings as we have not used any physical obstruction or other manipulations on the aorta or on the renal artery. Thus, there is no reason to believe that any changes in the aortic pressure would not be transmitted equally in the renal arteries that are directly branching out from the aorta. Although RAP was not measured directly from the renal arteries in the present study, MAP recorded from the carotid artery cannula with its tip advanced up to the arch of aorta and thus, the recorded MAP was representing the aortic pressure, thus equals to the RAP in in vivo mice preparations in the present study. The possibility of a reduction in RAP could cause such attenuation of UNaV response in TNFRKO mice is most unlikely as we do not see any statistical differences in MAP responses to AngII in these TNFRKO mice compared to those in WT mice. The consideration of RAP similar as aortic pressure has been considered in many studies in mice, rats and dogs from ours’ (Castillo et al., 2012; Majid & Navar, 1992; Shahid et al., 2008) as well as other laboratories (Brands & Hall, 1998; Gross et al., 1997; Mori & Cowley, 2004; Wei et al., 2020; Zhao & Navar, 2008). Thus, the marked attenuation of such natriuretic response in knockout mice, particularly in TNFR1KO mice compared to that in WT mice would not be attributed to any subtle difference, if any, in RAP responses in these mice.

In the present study in mice, we have not measured the level of TNF‐α during AngII infusion as it could not be possible to collect an additional amount of blood volumes required for this analysis during both the basal and AngII infusion periods. Earlier studies have demonstrated that TNF‐α mRNA and protein can be induced by AngII treatment in heart tissue within 30 min of administration (Kalra et al., 2002). We demonstrated earlier (Shahid et al., 2008) that the TNF‐α infusion that would achieve a plasma concentration of ~30 pmol, can induce natriuresis within 30 min of infusion which would be prevented by TNF‐α inhibitor, etanercept. In another study (Shahid et al., 2010), we have shown that systemic NO inhibition can induce TNF production that reached a plasma concentration of over 100 pg/ml within 60 min of NOS inhibitor, L‐NAME and such production of TNF‐α induces natriuresis which also could be prevented by etanercept. Thus, it is conceivable that AngII‐induced natriuresis can be attributed to TNF‐α release acting via TNFR1 as this natriuretic response is prevented in TNFR1KO mice (Castillo et al., 2012). It is to be mentioned here that such induction of TNF‐α release would be dose dependent as low doses of AngII usually induce antinatriuretic response.

AngII has stimulatory effects on sodium transport in multiple nephron segments via its binding with AT1 receptor (Mamenko et al., 2012; Satou et al., 2018; Zhao & Navar, 2008). It is reported that AngII enhances activity of ENaC in the aldosterone‐sensitive distal nephron (Mamenko et al., 2012; Zaika et al., 2013). In addition to its well‐described stimulatory actions on aldosterone secretion, AngII is also capable to directly increase ENaC activity (Mamenko et al., 2012; Zaika et al., 2013). However, we have also reported earlier that the natriuretic response to TNF‐α is linked to its direct inhibitory action on distal tubular ENaC activity in the kidney via its activation of TNFR1 receptors (Castillo et al., 2012; Majid, 2011; Shahid et al., 2008). As TNF‐α‐induced natriuretic response is mediated by TNFR1, the marked decreases in AngII‐induced natriuretic responses in TNFR1KO mice strongly suggest that such natriuretic response to a pressor dose of AngII is mediated by, at least in part, by the concomitant release of TNF‐α during AngII infusion. It is to be noted here that the expressions and activities of the tubular ion transporters during upregulation of TNF‐α has also not been carefully examined earlier. Thus, as the findings in the present study are warranted, future studies would be needed to examine the possible changes in the tubular ion transporters during TNF‐α generation during chronic elevated conditions of AngII.

In earlier studies in ours (Mehaffey et al., 2016; Mehaffey & Majid, 2017) and others’ laboratory (Chen et al., 2010), it has been observed that chronic AngII administration in TNFR1KO mice induced an exaggerated hypertensive response. These findings (Chen et al., 2010; Mehaffey et al., 2016) along with the results of the present study strongly suggest a role for TNFR1 in preventing hypertensive response to chronic elevation of AngII (Mehaffey & Majid, 2017). We have also shown that chronic AngII‐induced renal fibrotic and glomerulosclerotic lesion in WT mice was attenuated in TNFR2KO but not in TNFR1KO mice indicating that the renal inflammatory changes leading to renal injury induced by chronic ANG II administration are mainly mediated by TNFR2 but not TNFR1 (Singh et al., 2013). In the present study, we have not examined the role of TNFRs in other organs’ function except the kidney. Future systematic studies with comprehensive protocols would be needed to investigate the role of TNFRs in other organs during upregulation of TNF‐α during chronic elevation of RAS.

We have previously demonstrated that activation of TNFR1 but not TNFR2 resulted in natriuretic response to TNF‐α infusion in the kidney (Castillo et al., 2012). However, in the present study, it has been noted that the natriuretic response to AngII is also quantitatively less (though statistically insignificant) in TNFR2KO mice compared to WT mice. As it has been suggested that an activation of TNFR1 requires the coordinate expression of TNFR2 (Ardestani et al., 2013; Tartaglia et al., 1993), such partial reduction of AngII‐induced natriuretic response in TNFR2KO mice could be related to reduction in TNFR1 activity due to lack of TNFR2 in that strain of mice. Besides formation of heterocomplexes with TNFR1, TNFR2 also potentiate responses mediated by TNFR1 by increasing the local concentration of surface‐associated TNF‐α available to TNFR1 (Ardestani et al., 2013). This in vitro cellular study (Ardestani et al., 2013) has demonstrated that the binding of TNF‐α to TNFR2 serves to facilitate the binding of TNF‐α to TNFR1. Thus, it is conceivable that TNFR1‐induced natriuretic responses would be less in TNFR2KO mice compared to those in WT mice. It seems that both receptors may have some interdependent role in reducing the natriuretic response to acute AngII infusion which may be depend on the local concentration of TNF. Future comprehensive studies will be needed to understand more of this interdependency of TNF receptors in AngII‐induced responses.

We did not observe any significant changes in UKV in response to acute AngII infusion in these mice. Such findings in response to acute AngII infusion are also reported earlier in other studies in mice (Cervenka et al., 1999; Zhao & Navar, 2008). The reason is not known but it is to be mentioned that AngII administration will have variable effects on potassium excretion depending on its effect aldosterone secretion as well as on the concomitant changes in GFR, proximal tubular and collecting duct reabsorption (Giebisch, 1998). It could be related to a short period of AngII infusion as in the present study which would not have significant effect on aldosterone secretion and thus would have minimal effect on UKV. Anyway, there was a slight but significant decrease in UKV, when the UNaV response was attenuated markedly in TNFRKO mice that could be related to the changes in tubular sodium reabsorption (Giebisch, 1998).

## 5 CONCLUSION

The findings in the present investigation showed that TNF‐α receptors, particularly TNFR1 are involved generally in the natriuretic response that occurs during acute infusion of AngII and suggested that such activation of TNF‐α receptors is mediated by the concomitant generation of TNF‐α induced by AngII treatment. These data suggest a protective role for TNF‐α receptors, particularly TNFR1 in preventing excessive salt retention at clinical conditions associated with elevated renin–angiotensin system.

### 5.1 Perspective and significance

The findings in the present investigation that is the involvement of TNF‐α in the natriuretic response to elevated AngII level is very novel which is suggestive of a protective role for TNF‐α receptors, particularly TNFR1, by preventing excessive salt retention and thus, helps to minimize the chronic blood pressure elevation at the clinical conditions associated with enhancement of RAS (Battula et al., 2011; Chen et al., 2010; Majid et al., 2015; Mehaffey et al., 2016; Mehaffey & Majid, 2017). Thus, the pathophysiological significance of these findings needs to be explored further with alterations in RAS in chronic experimental conditions (Majid et al., 2015; Mehaffey & Majid, 2017). TNFα production induced by AngII appears in the circulation as its soluble form (sTNF‐α) which is formed by proteolytic cleavage by TNFα‐converting enzyme from its membrane tethered (mTNF‐α) isoform (Steed et al., 2003). While mTNF‐α has the equal affinity for both the receptors, the circulating sTNF‐α has the maximal high affinity to interact with TNFR1 with no or minimal affinity for TNFR2 YY (Steed et al., 2003). Thus, when there is increase in the circulating level of TNF‐α in response to any pathophysiologic stimulus, the TNFR1‐mediated renal functional changes would have a significant role in such clinical conditions.

## CONFLICT OF INTEREST

The authors have no conflict of interest, financial, or otherwise, relevant to this article.

## AUTHORS' CONTRIBUTION

DSAM designed the study and AC performed the experiments, collected, and analyzed data with statistical analysis. DSAM reviewed the processed data with statistical analysis, designed the final figures, and wrote the manuscript. Both the authors reviewed and revised the manuscript, approved the final manuscript as submitted, and agree to be accountable for all aspects of the work.
